# Mechanism of Reactions of 1-Substituted Silatranes and Germatranes, 2,2-Disubstituted Silocanes and Germocanes, 1,1,1-Trisubstituted Hyposilatranes and Hypogermatranes with Alcohols (Methanol, Ethanol): DFT Study

**DOI:** 10.3390/molecules25122803

**Published:** 2020-06-17

**Authors:** Denis Chachkov, Rezeda Ismagilova, Yana Vereshchagina

**Affiliations:** 1 Kazan Department of Joint Supercomputer Center of Russian Academy of Sciences–Branch of Federal State Institution “Scientific Research Institute for System Analysis of the RAS”, Lobachevskogo 2/31, 420111 Kazan, Russia; de2005c@gmail.com; 2 Department of Physical Chemistry, A.M. Butlerov Institute of Chemistry, Kazan Federal University, Kremlevskaya 18, 420008 Kazan, Russia; ism.rezeda@gmail.com

**Keywords:** atranes, ocanes, hypoatranes, conformational analysis, mechanism of reaction, alcohols, DFT calculations

## Abstract

The mechanism of reactions of silatranes and germatranes, and their bicyclic and monocyclic analogues with one molecule of methanol or ethanol, was studied at the Density Functional Theory (DFT) B3PW91/6-311++G(df,p) level of theory. Reactions of 1-substituted sil(germ)atranes, 2,2-disubstituted sil(germ)ocanes, and 1,1,1-trisubstituted hyposil(germ)atranes with alcohol (methanol, ethanol) proceed in one step through four-center transition states followed by the opening of a silicon or germanium skeleton and the formation of products. According to quantum chemical calculations, the activation energies and Gibbs energies of activation of reactions with methanol and ethanol are close, their values decrease in the series of atranes–ocanes–hypoatranes for interactions with both methanol and ethanol. The reactions of germanium-containing derivatives are characterized by lower activation energies in comparison with the reactions of corresponding silicon-containing compounds. The annular configurations of the product molecules with electronegative substituents are stabilized by the transannular N→X (X = Si, Ge) bond and different intramolecular hydrogen contacts with the participation of heteroatoms of substituents at the silicon or germanium.

## 1. Introduction

Heterocyclic compounds of hypervalent atoms, primarily intra-complex compounds of triethanolamine–metallatranes, possess unique physical and chemical properties that are due to their unusual trigonal-bipyramidal structure with a transannular nitrogen–element bond and the presence of an axial fragment. Triethanolamine derivatives of silicon, germanium, tin, carbon, boron, phosphorus, and arsenic, as well as bismuth, titanium, vanadium, aluminum and iron are currently known [1,2]. The most studied compounds of the elements of group 14 are silatranes and germatranes, as well as their bicyclic analogues–ocanes and monocyclic–hypoatranes. The diverse biological activity of atrane systems depends on the nature of the substituents, and such compounds can be both effective immunostimulants and adaptogens, and immunosuppressants, thus providing a stimulating or inhibiting effect on the vital activity of micro- and macroorganisms [3,4,5,6,7,8,9,10]. In addition, atranes are used as important functional reagents [11,12,13,14,15], catalysts for the formation of polyurethanes [16,17].

Recent achievements, challenges and prospects for using atranes are given in the reviews [18,19,20]. Atranes, ocanes and hypoatranes contain an intramolecular coordination bond nitrogen→element. Theoretical studies on the structure and hypervalent intramolecular coordination N→X (X = C, Si, Ge, B, P) in some atrane systems were carried out in [21,22,23,24,25,26,27,28,29,30,31]; information on the structure of some ocanes is given in [32,33,34,35,36,37,38,39], and hypoatranes in [36,40,41,42,43].

The length and strength of the intramolecular transannular bond N→X is determined by the number and nature of electronegative substituents at the central atom X: An increase in the number of highly electronegative substituents at the silicon or germanium atom significantly reduces the length of the N→X bond and, therefore, increases its strength [33].

Despite the long history of the chemistry of atrane systems, issues affecting their spatial and electronic structure, as well as the reaction mechanisms of these compounds, do not lose their relevance, and the development of quantum chemistry methods contributes a lot to this. These new results allow the available experimental data to be substantiated. Various aspects of the reactivity of atranes are considered in reviews [19,44], the study of the electron-donating ability of heteroatoms of the silatranyl group by calculation methods is described in [45,46,47]. There are a few publications concerned with reaction mechanisms of metallatranes and their bicyclic and tricyclic analogs with nucleophiles. The results of theoretical studies of the hydrolysis reactions of some atrane, ocane and hypoatrane systems are described in [22,43,48,49,50,51,52].

In the present work, we performed a theoretical study of the reactions of 1-substituted atranes, 2,2-disubstituted ocanes, and 1,1,1-trisubstituted hypoatranes with nucleophilic reagents—alcohols (methanol and ethanol)—and compared the results with the available data for hydrolysis reactions of these compounds.

## 2. Results and Discussion

### 2.1. Methodology

**Scheme 1:**
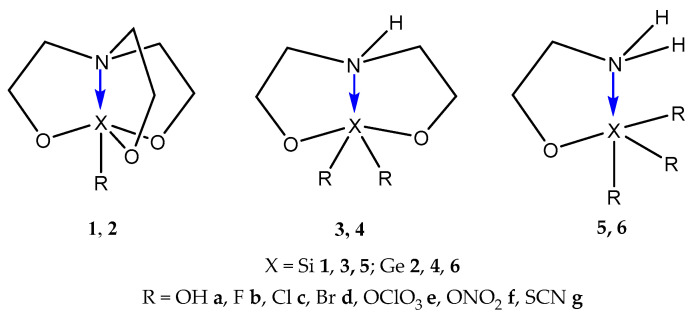
Structures of atranes, ocanes, and hypoatranes.

A theoretical study of the reactions of 1-substituted silatranes **1a**–**g** and germatranes **2a**–**g**, 2,2-disubstituted silocanes **3a**–**g** and germocanes **4a**–**g**, 1,1,1-trisubstituted hyposilatranes **5a**–**g** and hypogermatranes **6a**–**g** (Scheme 1) with nucleophilic reagents methanol and ethanol was carried out by quantum chemical calculations.

The Density Functional Theory (DFT) with B3PW91 hybrid functional [53,54] and 6-311++G(df,p) [55] extended basis set (calculations of the molecules in vacuum) using GAUSSIAN 09 software package [56] were earlier successfully used to study hydrolysis reactions of atrane, ocane and hypoatrane systems [43,52]. The choice of the B3PW91 method was also based on the data [57]. In all cases, the geometric parameters of the molecules were fully optimized. The critical points on the potential energy surfaces were identified as energy minima by the absence of imaginary frequencies in the corresponding Hessian matrices and as transition states by the presence of one imaginary frequency therein. Transition states were assigned to reaction paths by descent toward starting molecule and reaction product. Hessian was generated analytically in all cases, when searching for minimum energy it was calculated once to confirm that a minimum energy has been entered. In the case of a search for transition state, the Hessian was analytically calculated at each iteration. The parameter GRID = UltraFine was used. The solvent has not been taken into account.

Reactions of 1-substituted silabicyclo[3.3.3]undecanes **1a**–**g** and 1-germabicyclo[3.3.3]undecanes **2a**–**g**, 2,2-disubstituted 1,3,6,2-dioxazasilocanes **3a**–**g** and 1,3,6,2-dioxazagermocanes **4a**–**g**, hyposilatranes **5a**–**g** and hypogermatranes **6a**–**g** with one methanol molecule or one ethanol molecule were simulated.

First, we carried out theoretical conformational analysis of all starting compounds **1**–**6**. Namely, the conformational analysis of all reaction participants is a necessary step in studying their reactivity and establishing the reaction mechanisms. We found energetically preferred conformers for each of compounds **1**–**6**. These structures were used as the starting reagents in the simulation of the mechanism of their reactions with alcohol (methanol or ethanol). The energy difference between the conformers was 5–15 kJ/mol. The barrier of the transition between the conformers was noticeably higher: 18–46 kJ/mol. The reactions studied can proceed not only for the most favorable conformer of the starting reagent, but also for those which are not advantageous. The reaction barrier for unfavorable conformers was 5–25 kJ/mol higher than for the most favorable conformation of the reagent.

According to our previous quantum chemical calculations [52], 1-substituted compounds **1a**–**g**, **2a**–**g** are classical atrane systems where the silicon or germanium atom has a flattened tetrahedral configuration, and the nitrogen moiety is also flattened; the substituent R at the X atom occupies the axial position. Eight-membered silicon-nitrogen-oxygen-containing cycles in them have *crown* conformations (*chair–chair* or *chair–bath*), the molecules have a transannular N→X bond (X = Si, Ge).

The most preferred conformation of bicyclic analogues of atranes–2,2-disubstituted ocanes, **3a**–**g**, **4a**–**g**, is symmetrical *crown* (*chair–chair*) where the silicon (germanium) and nitrogen atoms are close, the transannular interaction N→X (X = Si, Ge) is observed [52]. The silicon and germanium atoms are tetrahedra with one axial and one equatorial substituent.

In the preferred conformers of monocyclic hypoatranes **5a**–**g**, **6a**–**g** [43], the silicon (germanium) and nitrogen atoms are flattened tetrahedra where oxygen atom and two substituents at the Si (Ge) are in equatorial positions, and third substituent is axial. In hypoatranes with perchlorate (**5e**, **6e**), nitrate (**5f**, **6f**), and thiocyanate (**5g**, **6g**) substituents, intramolecular contacts between one of the hydrogen atoms of the amino group and the heteroatom (O or N) of one of the functional groups at the Si and/or Ge, in halogen derivatives (**5b**–**d**, **6b**–**d**), contacts between the hydrogen atoms of the amino group and the halogen atoms are possible. The *Endo*-configuration of the molecules **5a**–**g**, **6a**–**g** is determined by the transannular interaction N→Si or N→Ge as well as for atranes **1a**–**g**, **2g** and ocanes **3a**–**g**, **4a**–**g**.

### 2.2. Reactions of 1-Substituted Atranes with Methanol

**Scheme 2:**
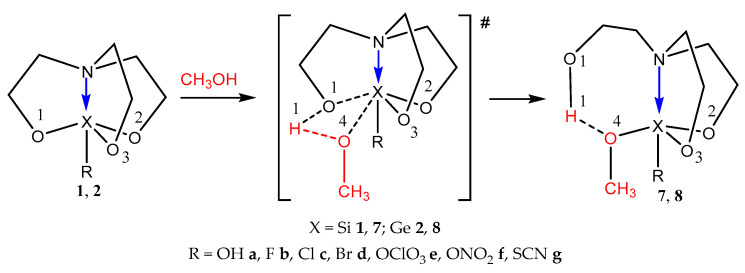
Mechanism of reactions of atranes with methanol.

**Figure 1:**
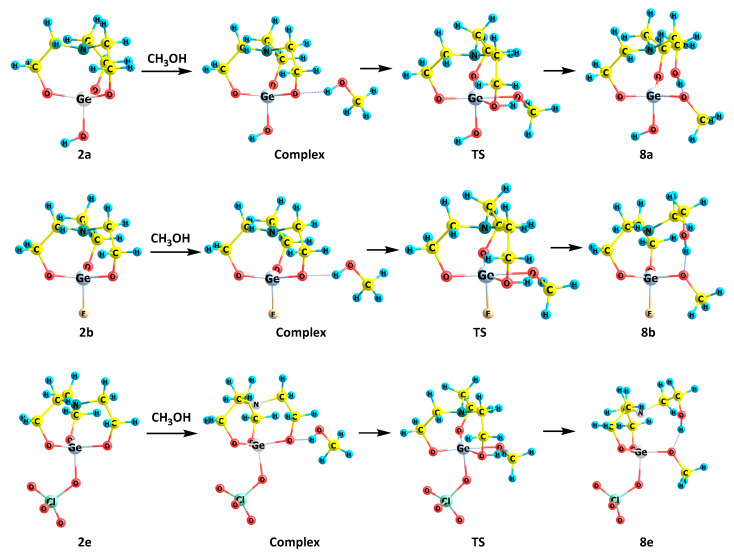
Mechanism of reactions of germatranes **2a**, **2b**, **2e** with methanol according to DFT calculations.

The mechanism of reactions of atranes **1a**–**g**, **2a**–**g** with one methanol molecule according to quantum chemical calculations is shown in Scheme 2. The obtained energy characteristics of the reactions and some geometric parameters of the reaction participants are given in Table 1 and Table S1. As an example, the reaction schemes for some atranes according to theoretical studies are presented in Figure 1.

The interaction of atranes **1a–g**, **2a–g** with methanol proceeds in one step. Initially, prereaction complexes are formed between the molecules of atrane and methanol, in which the N→Si (N→Ge) bond length decreases, a contact arises between the proton of the hydroxyl group of methanol and the O1 atom of the atrane skeleton (Table S1). The prereaction complexes are transformed into transition states in which the N→Si (N→Ge) distance is further reduced, the Si(Ge)‒O1 and O4‒H1 bonds are stretched in methanol, and the H1^…^O1 contact is reduced (Scheme 2, Table S1). The third stage of interaction is the opening of one of the half rings of the atrane skeleton, as a result, the Si(Ge)‒O1 bond is cleaved, and the Si(Ge)‒O4 and O1‒H1 bonds are formed. The products of methanol addition to atranes are substituted ocanes—2-R-6-(2-hydroxyethyl)-1,3,2,6-dioxazasil(germ)ocane-2-ols **7a–g**, **8a–g**.

According to the quantum chemical calculations, the possible intramolecular hydrogen bonds O4^…^H1O1 as well as the transannular interactions N→Si or N→Ge (Scheme 2, Table S1, and Figure 1) stabilize the structure of molecules **7a–g**, **8a–g**. The ocane fragments of product molecules have *crown* conformation—a *chair–boat* for silocane **7a** and germocanes **8a**–**g**, and symmetrical *boat–boat* for silocanes **7b**–**g**. The silicon (germanium) atom is flattened tetrahedron, and the nitrogen-containing fragment is also flattened. The N→Si (N→Ge) bond in the molecules of products **7a**–**g**, **8a**–**g** is longer (Table S1) than in the molecules of the starting atranes, which is generally consistent with the change in the N→Si (N→Ge) bond length in similar atranes and their hydrolysis products [52]. In the series of halogen-substituted products **7b**–**d** and **8b**–**d**, the distance N→Si (N→Ge) increases from F to Br. The smallest distance is observed for perchlorate derivatives **7e** and **8e**. The intramolecular hydrogen bonds O4^…^H1O1 in molecules **7a**–**g**, **8a**–**g** are quite strong, as evidenced by their lengths (Table S1); the strongest ones are in hydroxyl **8a** and fluorine **8b** derivatives, moreover the angle O4H1O1 is 161°–176°.

The analysis of Table 1 shows that the reactions of compounds **1a**–**g**, **2a**–**g** with methanol are controlled by thermodynamic factors; the contribution of the enthalpy component is most significant, since the activation enthalpy and the Gibbs activation energy change symbiotically. This conclusion is valid not only for activation parameters, but also for the enthalpy of the reaction (thermal effect) and for the Gibbs energy of the reaction.

In the series of halogenated silatranes and germatranes (F, Cl, Br), the activation energy increases (Table 1) naturally with a decrease in the electronegativity of the substituents [33,58]. The reactions of germatranes **2a**–**g** proceed with slightly lower activation energies compared with reactions of silatranes **1a**–**g**. In transition states, the smallest distance N‒X (X = Si, Ge) and the lowest activation energy (Table 1, Table S1) are observed for perchlorate-substituted atranes **1e** and **2e**.

### 2.3. Reactions of 2,2-Disubstituted Ocanes with Methanol

**Scheme 3:**
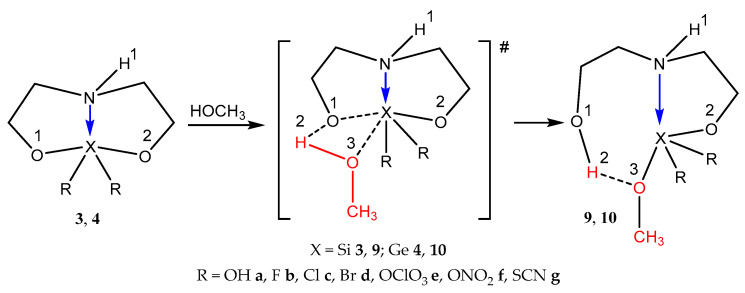
Mechanism of reactions of ocanes with methanol.

**Figure 2:**
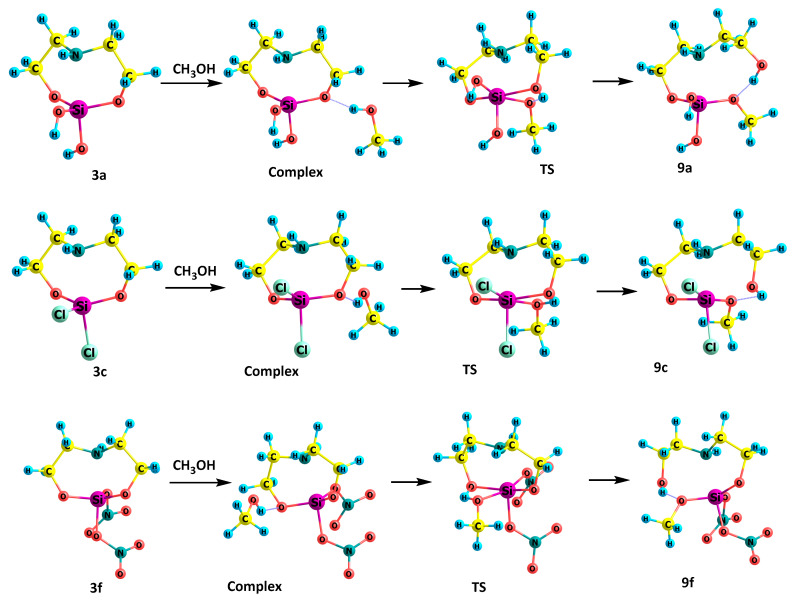
Mechanism of reactions of silocanes **3a**, **3c**, **3f** with methanol according to DFT calculations.

According to DFT calculations, the mechanism of reactions of ocanes **3a**–**g**, **4a**–**g** with one methanol molecule is presented in Scheme 3. The obtained energy characteristics of the reactions and some geometric parameters of the reaction participants are given in Table 2 and Table S2. As an example, the reaction schemes for some ocanes according to theoretical studies are presented in Figure 2.

The interaction of ocanes **3a**–**g**, **4a**–**g** with methanol also proceeds in one step. Initially, prereaction complexes are formed between the molecules of ocanes and methanol, in which the N‒Si (N‒Ge) bond length decreases, a contact arises between the proton of the hydroxyl group of methanol and the O1 atom. Prereaction complexes are transformed into transition states, in which N‒Si (N‒Ge) distance is further reduced, while the Si‒O1 (Ge‒O1) bond is extended (Table S2), the silicon(germanium)- and nitrogen-containing fragments are flattened, which leads to distortion of *crown* conformation. The O3‒H2 bond is stretched, and the O1‒H2 contact arises.

The nucleophilic attack of a methanol molecule leads to the cleavage of the Si‒O1 (Ge‒O1) bond in the molecules of ocanes **3a**–**g**, **4a**–**g** and the appearance of a new Si‒O3 (Ge‒O3) bond. The annular configuration of product molecules **9a**–**g**, **10a**–**g** is stabilized due to the dative interaction N→Si (N→Ge) and the intramolecular hydrogen bond O2^…^H3 (Table S2). The possibility of a transannular interaction N→Si (N→Ge) is evidenced by both the spatial factor (the distance N‒Si (N‒Ge) is less than the sum of the van der Waals radii of these elements) and the presence of electronegative substituents at the acceptor silicon or germanium atom. In addition, short contacts between the hydrogen (N‒H) and the heteroatom of the axial substituent at the Si (Ge) atom contribute to the stabilization of the *crown*-like 10-membered configuration of molecules: oxygen in hydroxyl substituted **9a** and **10a**, perchlorate **9e** and **10e**, nitro **9f** and **10f**, halogen in **9b**–**d** and **10b**–**d**, nitrogen of the thiocyanate group in **9g** and **10g**.

The silicon, germanium and nitrogen atoms in the molecules **9a**–**g**, **10a**–**g** are flattened tetrahedra. The N–Si (N‒Ge) distance in the molecules of the products **9a**–**g**, **10a**–**g** is less than in the reagents, as well as in the hydrolysis reactions of these compounds [52], with the exception of dichloro- and dibromo-substituted germocanes **10c** and **10d** (Table S2). Silocanes are characterized by a regular decrease in the N–X distance as the electronegativity of the exocyclic halogen substituent increases in a series—F, Cl, Br; however, the opposite regularity is observed for germocanes (Table S2).

The reactions of ocanes **3a**–**g**, **4a**–**g** are controlled by thermodynamic factors. The activation energy in a series of halogen-substituted ocanes **9b**–**d** and **10b**–**d** (F, Cl, Br) increases naturally with a decrease in the electronegativity of the substituents (Table 2). In the case of reactions of germocanes **4a**–**g**, the activation energies and Gibbs activation energies are lower than those of silocanes **3a**–**g**. The smallest N–Si (N‒Ge) distances and the smallest values of the activation energy (Gibbs activation energy) are observed for the diperchlorate derivatives **9e** and **10e** (Table 2, Table S2). The same regularity was revealed for hydrolysis reactions of similar ocanes [52].

### 2.4. Reactions of 1,1,1-Trisubstituted Hypoatranes with Methanol

**Scheme 4:**
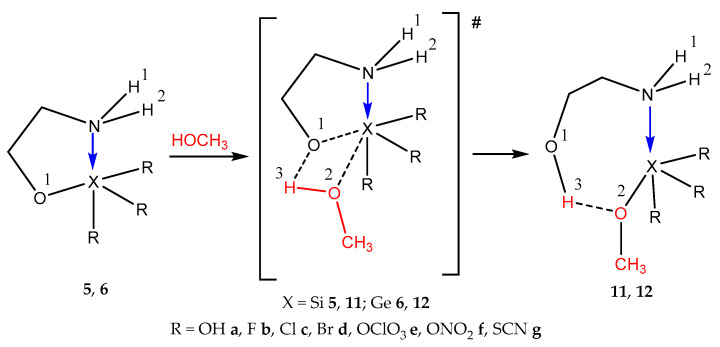
Mechanism of reactions of hypoatranes with methanol.

**Figure 3:**
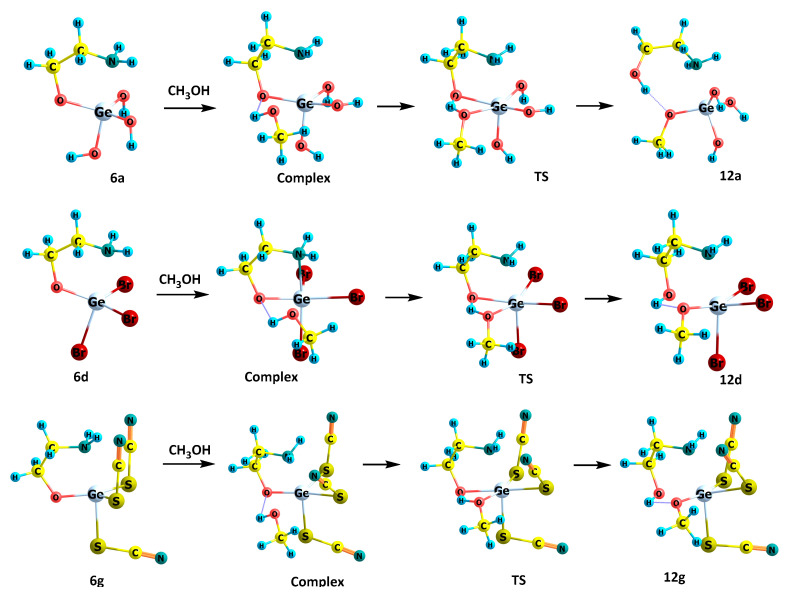
Mechanism of reactions of hypogermatranes **6a**, **6d**, **6g** with methanol according to DFT calculations.

The mechanism of reactions of 1,1,1-substituted hyposilatranes **5a**–**g** and hypogermatranes **6a**–**g** with one methanol molecule according to quantum chemical calculations is shown in Scheme 4. The obtained energy characteristics of the reactions and some geometric parameters of the reaction participants are given in Table 3 and Table S3. As an example, the reaction schemes for some ocanes according to theoretical studies are presented in Figure 3.

The interaction of hypoatranes **5a**–**g, 6a**–**g** with methanol proceeds in several stages, as in the case of atranes **1a**–**g, 2a**–**g** and ocanes **3a**–**g, 4a**–**g**. At the first stage, prereaction complexes are formed in which the reagent molecules come together, the distance N‒X (X = Si, Ge) is reduced (Scheme 4, Table S3). Then, the prereaction complexes are transformed into transition states, the distances N‒X (X = Si, Ge) and O1^…^H3 are shortened, and the X‒O1 bond is stretched, a new contact arises between the silicon (germanium) atom and the oxygen atom of methanol (Scheme 4, Table S3). At the final stage, the Si‒O1 (Ge‒O1) bond breaks and a new Si‒O2 (Ge‒O2) bond arises, resulting in reaction products–complexes of 2-aminoethanol with trisubstituted methoxysilanes **11a**–**g** and methoxygermanes **12a**–**g**, in which the N–Si or N‒Ge distance is shorter than in the reagent molecules (Table S3).

The configuration of molecules **11a**–**g, 12a**–**g** is due to the transannular bond N→Si or N→Ge, the dative interaction O→Si or O→Ge (**11b**–**g**, **12b**–**g**), and the bonding between the oxygen of methoxyl substituent at the Si (Ge) and a hydrogen of the hydroxyl group of 2-aminoethanol (with the exception of trinitroderivative **12f**) (Scheme 4, Table S3). In addition, as in the starting molecules **5b**–**g** and **6b**–**g**, in complexes **11b**–**g** and **12b**–**g** hydrogen contacts between the hydrogen atoms of the amino group and heteroatoms (halogen, oxygen of perchlorate or nitro groups, and nitrogen of thiocyanate groups) of substituents at the Si or Ge are possible. In complexes **11a**–**g** and **12a**–**g**, the silicon or germanium polyhedron expands: The tetravalent Si or Ge atom is six-coordinated and has the structure of a distorted tetragonal bipyramid. Two substituents at the silicon (germanium) and oxygen atoms lie in the equatorial plane of this bipyramid, and the nitrogen and third substituent at the Si (Ge) occupy axial positions (Scheme 4, Figure 3). Apparently, the preferred configurations of the complexes **11a**–**g, 12a**–**g** are stabilized precisely due to the dative interactions and intramolecular hydrogen bonds arising because of the favorable orientation of the substituents at the silicon or germanium.

The reactions of hypoatranes **5a**–**g, 6a**–**g** with methanol, as in the case of ocanes **3a**–**g, 4a**–**g** and atranes **1a**–**g, 2a**–**g**, are controlled by thermodynamic factors. In series of halogen-substituted (F, Cl, Br) hyposilatranes and hypogermatranes, the activation energy increases (Table 3), which is naturally associated with a decrease in the electronegativity of the substituents and is consistent with the data for the corresponding atranes and ocanes. For the reactions of hypogermatranes **6a**–**g**, the activation energies are lower in comparison with the reactions of hyposilatranes **5a**–**g** (Table 3). The smallest N‒X (X = Si, Ge) distance in the transition states and the lowest activation energy are observed for the reactions of triperchlorate derivatives **5e** and **6e** (Table 3, Table S3).

**Figure 4:**
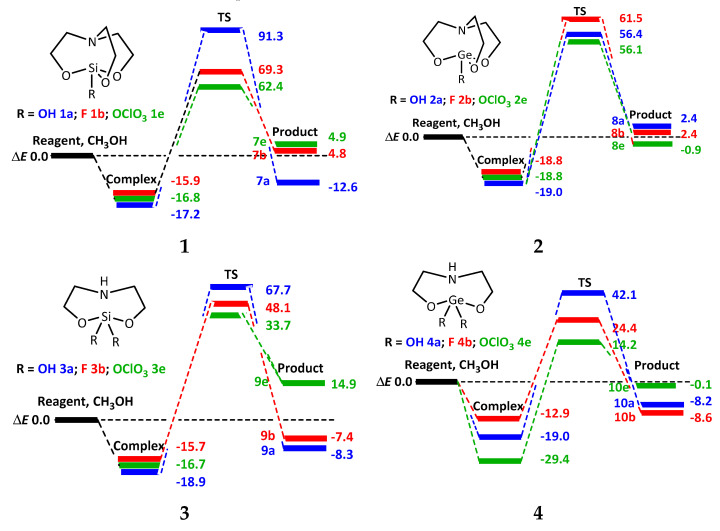

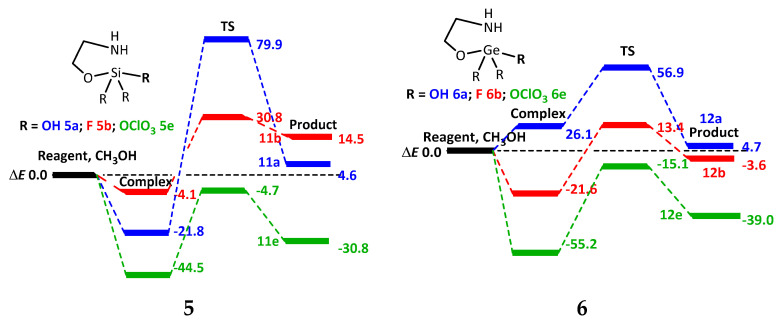
Energy diagrams for reactions of **1a**,**b**,**e**; **2a**,**b**,**e**; **3a**,**b**,**e**; **4a**,**b**,**e**; **5a**,**b**,**e**; **6a**,**b**,**e** with methanol (Δ*E*, kJ/mol).

The energy diagrams of the reactions of hydroxy-, fluoro-, and perchlorate-substituted atranes, ocanes, and hypoatranes are shown in Figure 4. To estimate the thermal effect of the reactions, for zero we chose the energies of the starting reagent and methanol molecules infinitely distant from each other.

A comparison of the results obtained for 1-substituted atranes, 2,2-disubstituted ocanes and 1,1,1-trisubstituted hypoatranes (Table 1, Table 2 and Table 3) shows that the values of activation energy and Gibbs activation energy decrease in the series of atranes—ocanes—hypoatranes. A decrease in the number of cycles in the molecule in this series and an increase in the number of highly electronegative substituents (having negative mesomeric and inductive effects) at the silicon or germanium atom led to a decrease in the N‒Si or N‒Ge bond length (Tables S1–S3) and, consequently, an increase in its strength. Probably, this fact, along with the presence of dative and intramolecular hydrogen interactions, explains the high stability of trisubstituted hypoatranes obtained in an aqueous–alcoholic medium at elevated temperatures [36,40,41,42].

### 2.5. Reactions of Atranes, Ocanes, and Hypoatranes with Ethanol

**Scheme 5:**
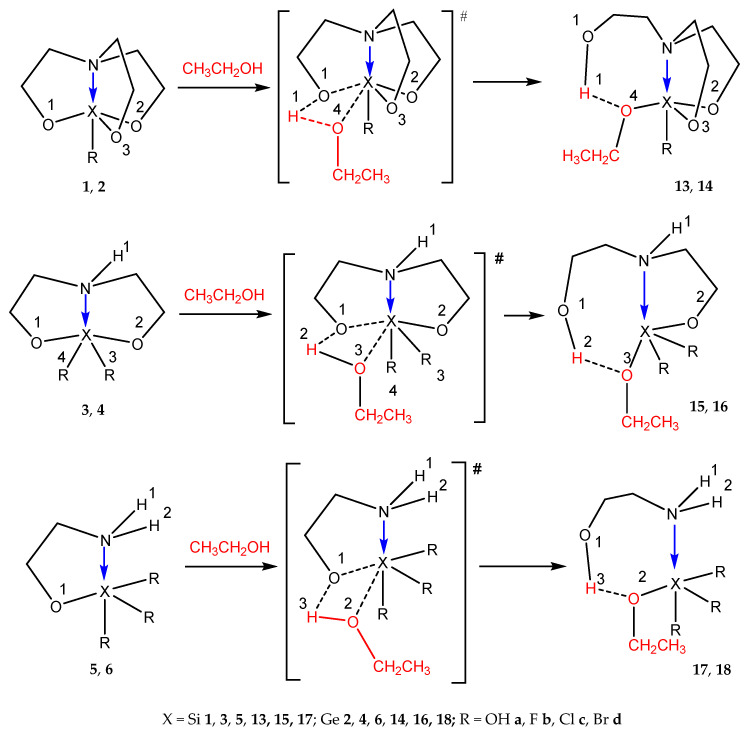
Mechanism of reactions of atranes, ocanes, and hypoatranes with ethanol.

We studied the reactions of substituted atranes and their analogues with ethanol using the reactions of hydroxy derivatives **1a, 2a, 3a, 4a, 5a, 6a** and halogen derivatives **1b**–**d, 2b**–**d**, **3b**–**d**, **4b**–**d**, **5b**–**d**, **6b**–**d** to compare the interactions with methanol. The mechanism of reactions of 1-substituted silatranes and germatranes, 2,2-disubstituted silocanes and germocanes, 1,1,1-trisubstituted hyposilatranes and hypogermatranes with one ethanol molecule according to quantum chemical calculations is shown in Scheme 5. The calculated data are given in Table 4, Tables S4–S6.

As in the case of interaction with methanol, the reactions with ethanol proceed in one step: Initially, prereaction complexes are formed that transform into transition states, as a result of the cleavage of one Si‒O (Ge‒O) bond and the arising of a new Si‒O(CH_2_CH_3_) or Ge‒O(CH_2_CH_3_) bond, the reaction products are formed.

The transition states are characterized by a decrease in the N‒Si (N‒Ge) distance, the Si(Ge)‒O1 and O‒H bonds are stretched, and the H1^…^O1 contact is reduced (Scheme 5, Tables S4–S6). Molecules of the products of ethanol addition reactions have a configuration similar to the configuration of the corresponding product molecules formed upon interaction with methanol.

The products of reactions of atranes are substituted silocanes **13a**–**d** and germocanes **14a**–**d**. According to the quantum chemical calculations, the ocane fragments of product molecules have *crown* conformation—symmetrical *boat–boat* for silocanes and *chair–boat* for germocanes. The silicon (germanium) and nitrogen-containing fragments are flattened. The transannular interaction N→Si or N→Ge and the possible intramolecular hydrogen bonds O4^…^H1O1 stabilize these structures (Table S4, Scheme 5). The N→Si (N→Ge) bond in the molecules of products **13a**–**d** and **14a**–**d** is longer than in the molecules of the starting atranes (Table S4).

The annular configuration of product molecules **15a**–**d** and **16a**–**d** is also stabilized due to the dative interaction N→Si (N→Ge) and the intramolecular hydrogen bond O2^…^H3 (Table S5, Scheme 5). In addition, short contacts between the hydrogen atom at the nitrogen and the heteroatom of the axial substituent at the silicon (germanium) atom contribute to the stabilization of the *crown*-like 10-membered configuration of molecules **15a**–**d** and **16a**–**d**: oxygen in the hydroxy-substituted **15a** and **16a** or halogen in **15b**–**d** and **16b**–**d**. The N‒Si (N‒Ge) distance in the molecules of products **15a**–**d** and **16a**–**d** is shorter than in the molecules of the starting ocanes, as in the case of reactions with methanol, and this distance increases in **15b** and **16d** (Table S5).

The configuration of the molecules **17a**–**d** and **18a**–**d** is due to the transannular interaction N→Si or N→Ge and hydrogen bonding O2^…^H3 between oxygen atom of the ethoxyl substituent at the silicon (germanium) and hydrogen atom of the hydroxy group of 2-aminoethanol (Scheme 5, Table S6). In addition, as in the starting molecules **5a**–**d** and **6a**–**d**, hydrogen contacts between the hydrogen atoms of the amino group and the heteroatoms (oxygen or halogen) of the substituents at the silicon or germanium are possible in products **17a**–**d** and **18a**–**d**. In complexes **17a**–**d** and **18a**–**d**, the tetravalent six-coordinated silicon or germanium atom has the structure of a distorted tetragonal bipyramid, in the equatorial plane of which there are two substituents at the silicon or germanium and oxygen atoms, and the nitrogen and the third substituent at the Si (Ge) occupy the axial positions.

The transannular interaction N→Si (N→Ge) in the product molecules is due to both the spatial factor and the presence of electronegative substituents at the acceptor silicon or germanium atom. In all product molecules, sufficiently strong hydrogen bonds are formed between the oxygen atom of the ethoxyl substituent at the silicon (germanium) and the hydrogen atom of the hydroxy group (Scheme 5, Tables S4–S6).

**Figure 5:**
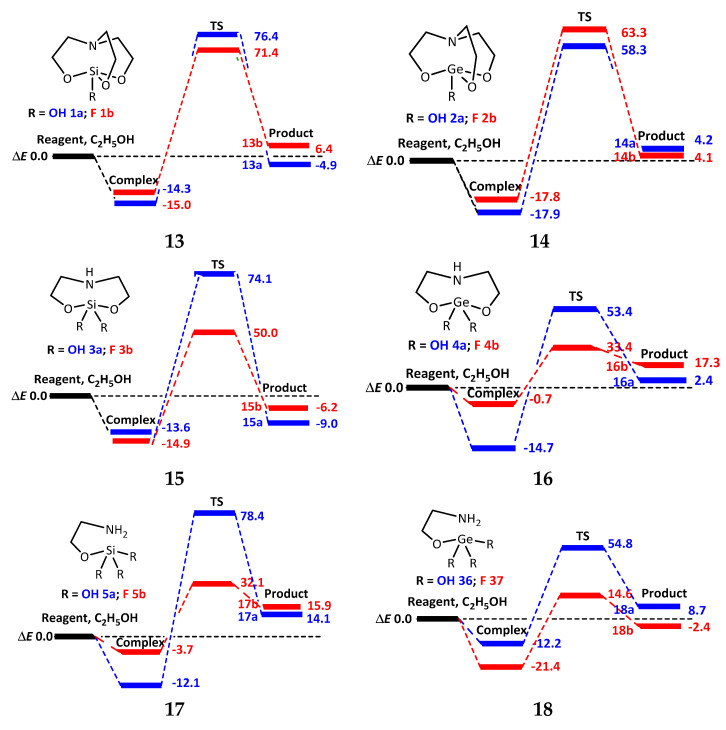
Energy diagrams of formation of products **13a**,**b**; **14a**,**b**; **15a**,**b**; **16a**,**b**; **17a**,**b**; **18a**,**b**.

The analysis of obtained data shows that the reactions with ethanol are controlled by thermodynamic factors (Table 4, Figure 5). The value of activation energy naturally increases in the series of halogen-substituted (F, Cl, Br) compounds **1b**–**d**, **2b**–**d**, **3b**–**d**, **4b**–**d**, **5b**–**d**, and **6b**–**d** by a decrease in the electronegativity of the substituents [33,58]. Reactions of germanium-containing derivatives proceed with slightly lower activation energies as compared with reactions of silicon-containing derivatives. In addition, the activation energy decreases in the series of atranes–ocanes–hypoatranes (Table 4, Figure 5). The same regularities were revealed for hydrolysis reactions of similar atranes, ocanes [52], and hypoatranes [43].

A comparison of the results for reactions of atranes, ocanes and hypoatranes with ethanol and the corresponding data for reactions with methanol allowed us to conclude that the activation energies are close; moreover, in the case of ocanes they are slightly higher in reactions with ethanol.

## 3. Conclusions

Thus, we have studied the mechanism of reactions of 1-sustituted sil(germ)atranes, 2,2-disustituted sil(germ)ocanes, and 1,1,1-trisubstituted hyposil(germ)atranes with one molecule of methanol or ethanol at the DFT B3PW91/6-311++G(df,p) level of theory. These reactions with alcohol (methanol, ethanol), as well as with water [43,52], proceed in one step through four-center prereaction complexes and transition states followed by the opening of heteroatom (silicon or germanium) skeleton and the formation of products.

According to DFT calculations, the activation energies and Gibbs energies of activation of reactions with methanol and ethanol are close, their values decrease in the series of atranes-ocanes-hypoatranes for reactions with both methanol and ethanol. Reactions of germanium-containing derivatives are characterized by lower activation energies in comparison with silicon-containing compounds.

The results of theoretical conformational analysis show that the annular configurations of the product molecules with electronegative substituents are stabilized by the transannular N→X (X = Si, Ge) interaction and different intramolecular hydrogen contacts with the participation of heteroatoms of substituents at the silicon or germanium.

## Figures and Tables

**Table 1:** Activation energies, Gibbs activation energies (kJ/mol), and imaginary vibration frequencies (cm^−1^) of transition states **^#^** of reactions of atranes **1a**–**g**, **2a**–**g** with methanol.

	Δ*E* ^#^	Δ*G* ^#^	ν_1_, cm^−1^
**1a**	91.3	149.4	877i
**1b**	69.3	128.1	910i
**1c**	72.4	131.9	951i
**1d**	73.2	132.8	966i
**1e**	62.4	120.4	992i
**1f**	71.9	129.1	969i
**1g**	78.8	135.8	972i
**2a**	56.4	113.2	821i
**2b**	61.5	115.6	803i
**2c**	67.3	121.4	815i
**2d**	68.7	122.7	819i
**2e**	56.1	110.9	891i
**2f**	66.3	119.4	851i
**2g**	72.4	124.8	822i

**Table 2:** Activation energies, Gibbs activation energies (kJ/mol), and imaginary vibration frequencies (cm^−1^) of transition states **^#^** of reactions of ocanes **3a**–**g**, **4a**–**g** with methanol.

	Δ*E* ^#^	Δ*G* ^#^	ν_1_, cm^−1^
**3a**	67.7	125.6	835i
**3b**	48.1	106.0	942i
**3c**	55.1	114.0	990i
**3d**	55.8	113.4	1020i
**3e**	33.7	93.3	1041i
**3f**	48.6	104.6	1000i
**3g**	49.9	107.6	1035i
**4a**	42.1	97.3	802i
**4b**	24.4	79.8	922i
**4c**	38.0	93.9	917i
**4d**	41.5	97.3	928i
**4e**	14.2	70.8	1010i
**4f**	37.6	92.6	950i
**4g**	38.5	95.3	952i

**Table 3:** Activation energies, Gibbs activation energies (kJ/mol), and imaginary vibration frequencies (cm^−1^) of transition states **^#^** of reactions of hypoatranes **5a**–**g**, **6a**–**g** with methanol.

	Δ*E* ^#^	Δ*G* ^#^	ν_1_, cm^−1^
**5a**	79.9	136.0	963i
**5b**	30.8	−95.1	1020i
**5c**	35.5	92.9	1049i
**5d**	39.3	96.3	1064i
**5e**	−4.7	56.0	1145i
**5f**	13.9	72.6	1074i
**5g**	28.4	83.9	1103i
**6a**	56.9	110.7	925i
**6b**	13.4	68.3	1000i
**6c**	27.7	84.2	995i
**6d**	33.4	89.9	994i
**6e**	−15.1	44.8	1120i
**6f**	3.6	61.2	1037i
**6g**	21.7	79.2	1034i

**Table 4:** Activation energies, Gibbs activation energies (kJ/mol), and imaginary vibration frequencies (cm^−1^) of transition states **^#^** of reactions of **1a**–**d**—**6a**–**d** with ethanol.

	Δ*E* ^#^	Δ*G* ^#^	ν_1_, cm^−1^
**1a**	76.4	137.4	893i
**1b**	71.4	130.5	917i
**1c**	74.8	134.5	956i
**1d**	75.7	134.7	973i
**2a**	58.3	115.5	833i
**2b**	63.3	117.9	814i
**2c**	69.5	124.1	820i
**2d**	71.1	125.6	828i
**3a**	74.1	130.9	857i
**3b**	50.0	108.1	951i
**3c**	57.1	116.1	998i
**3d**	57.9	116.5	1028i
**4a**	53.4	107.9	905i
**4b**	33.4	88.3	976i
**4c**	43.4	99.1	968i
**4d**	47.6	103.1	965i
**5a**	78.4	131.9	914i
**5b**	32.1	88.8	1030i
**5c**	36.8	93.6	1056i
**5d**	40.6	97.4	1072i
**6a**	54.8	106.6	883i
**6b**	14.6	69.7	1012i
**6c**	29.3	86.1	1003i
**6d**	35.2	91.9	1001i

## Supplementary Materials

The following are available online, Table S1: Selected interatomic distances (Å) in the reagents, prereaction complexes, transition states, and products of the reactions of atranes **1a**–**g, 2a**–**g** (**a** OH, **b** F, **c** Cl, **d** Br, **e** OClO_3_, **f** ONO_2_, **g** SCN) with methanol. For X‒N distances the Mayer bond order in the NAO basis are given in parentheses, Table S2: Selected interatomic distances (Å) in the reagents, prereaction complexes, transition states, and products of the reactions of ocanes **3a**–**g, 4a**–**g** (**a** OH, **b** F, **c** Cl, **d** Br, **e** OClO_3_, **f** ONO_2_, **g** SCN) with methanol. For X‒N distances the Mayer bond order in the NAO basis are given in parentheses, Table S3: Selected interatomic distances (Å) in the reagents, prereaction complexes, transition states, and products of the reactions of hypoatranes **5a**–**g, 6a**–**g** (**a** OH, **b** F, **c** Cl, **d** Br, **e** OClO_3_, **f** ONO_2_, **g** SCN) with methanol. For X‒N distances the Mayer bond order in the NAO basis are given in parentheses, Table S4: Selected interatomic distances (Å) in the reagents, prereaction complexes, transition states, and products of the reactions of atranes **1a**–**d, 2a**–**d** (**a** OH, **b** F, **c** Cl, **d** Br) with ethanol, Table S5: Selected interatomic distances (Å) in the reagents, prereaction complexes, transition states, and products of the reactions of ocanes **3a**–**d**, **4a**–**d** (**a** OH, **b** F, **c** Cl, **d** Br) with ethanol, Table S6: Selected interatomic distances (Å) in the reagents, prereaction complexes, transition states, and products of the reactions of hypoatranes **5a**–**d**, **6a**–**d** (**a** OH, **b** F, **c** Cl, **d** Br) with ethanol.
