# Supplementary material for: Mechanism of Reactions of 1-Substituted Silatranes and Germatranes, 2,2-Disubstituted Silocanes and Germocanes, 1,1,1-Trisubstituted Hyposilatranes and Hypogermatranes with Alcohols (Methanol, Ethanol): DFT Study
Source: Molecules. 2020 Jun 17;25(12):2803. doi: 10.3390/molecules25122803 (PMC7356647; doi:10.3390/molecules25122803)
Supplement: Supplementary file 1 [file molecules-25-02803-s001.pdf]

Table S1. Selected interatomic distances (Å) in the reagents, prereaction complexes, transition states, and products of the reactions of atranes **1a-g**, **2a-g** (**a** OH, **b** F, **c** Cl, **d** Br, **e** $OClO_3$, **f** ONO_2, **g** SCN) with methanol. For X–N distances the Mayer bond order in the NAO basis are given in parentheses.

	$X–N^{reagent}$	$X–N^{complex}$	$X–N^{TS}$	$X–N^{product}$	$X–O1^{TS}$	$X–O1^{product}$	$X–O4^{TS}$	$X–O4^{product}$	$O1–H1^{TS}$	$O1–H1^{product}$	$O4–H1^{TS}$	$O4–H1^{product}$
**1a**	2.463 (0.1258)	2.405 (0.1496)	2.198 (0.2594)	3.053 (0.0286)	1.957	4.004	2.031	1.643	1.203	0.972	1.194	1.839
**1b**	2.344 (0.1673)	2.323 (0.1806)	2.120 (0.2832)	2.521 (0.1246)	1.920	3.709	1.913	1.658	1.186	0.972	1.219	1.793
**1c**	2.382 (0.1595)	2.359 (0.1727)	2.136 (0.2733)	2.689 (0.0854)	1.914	3.773	1.913	1.650	1.196	0.971	1.207	1.814
**1d**	2.378 (0.1626)	2.354 (0.1761)	2.133 (0.2708)	2.981 (0.0389)	1.912	3.954	1.911	1.636	1.200	0.970	1.204	1.884
**1e**	2.210 (0.2308)	2.191 (0.2442)	2.077 (0.3030)	2.279 (0.2276)	1.898	3.605	1.880	1.653	1.209	0.970	1.198	1.823
**1f**	2.248 (0.2128)	2.232 (0.2252)	2.097 (0.2942)	2.362 (0.1918)	1.909	3.633	1.890	1.653	1.209	0.971	1.196	1.811
**1g**	2.240 (0.2179)	2.223 (0.2307)	2.100 (0.2878)	2.400 (0.1822)	1.909	3.665	1.911	1.658	1.208	0.970	1.193	1.826
**2a**	2.343 (0.1997)	2.321 (0.2134)	2.251 (0.2691)	2.423 (0.1932)	2.090	3.730	2.031	1.794	1.196	0.976	1.204	1.747
**2b**	2.280 (0.2220)	2.259 (0.2357)	2.212 (0.2798)	2.316 (0.2326)	2.065	3.743	2.031	1.790	1.180	0.976	1.222	1.747
**2c**	2.315 (0.2155)	2.291 (0.2298)	2.235 (0.2746)	2.386 (0.2120)	2.070	3.769	2.053	1.793	1.184	0.975	1.215	1.758
**2d**	2.322 (0.2135)	2.297 (0.2276)	2.239 (0.2719)	2.407 (0.2051)	2.074	3.772	2.060	1.794	1.186	0.974	1.212	1.762
**2e**	2.210 (0.2578)	2.193 (0.2688)	2.179 (0.2960)	2.224 (0.2777)	2.040	3.677	2.003	1.778	1.196	0.974	1.206	1.778
**2f**	2.242 (0.2446)	2.223 (0.2566)	2.200 (0.2872)	2.251 (0.2639)	2.051	3.697	2.022	1.782	1.191	0.975	1.208	1.763
**2g**	2.254 (0.2434)	2.237 (0.2551)	2.212 (0.2863)	2.292 (0.2513)	2.069	3.756	2.062	1.797	1.190	0.974	1.204	1.768

Table S2. Selected interatomic distances (Å) in the reagents, prereaction complexes, transition states, and products of the reactions of ocanes **3a-g**, **4a-g** (**a** OH, **b** F, **c** Cl, **d** Br, **e** $OClO_3$, **f** ONO_2, **g** SCN) with methanol. For X–N distances the Mayer bond order in the NAO basis are given in parentheses.

	X–N^{reagent}	X–N^{complex}	X–N^{TS}	X–N^{product}	X–O1^{TS}	X–O1^{product}	X–O3^{TS}	X–O3^{product}	O1–H2^{TS}	O1–H2^{product}	O3–H2^{TS}	O3–H2^{product}
**3a**	2.271 (0.2137)	2.227 (0.2372)	2.032 (0.3366)	2.231 (0.2399)	1.940	3.683	1.946	1.671	1.167	0.970	1.238	1.847
**3b**	2.262 (0.2170)	2.207 (0.2470)	2.043 (0.3322)	2.195 (0.2562)	1.919	3.687	1.892	1.660	1.190	0.969	1.222	1.877
**3c**	2.344 (0.2080)	2.257 (0.2478)	2.050 (0.3304)	2.042 (0.3234)	1.910	2.206	1.885	1.732	1.196	0.973	1.218	1.922
**3d**	2.318 (0.2223)	2.236 (0.2582)	2.044 (0.3250)	2.033 (0.3183)	1.907	2.181	1.880	1.728	1.203	0.972	1.211	1.941
**3e**	2.109 (0.3193)	2.067 (0.3411)	1.984 (0.3827)	1.967 (0.3732)	1.866	1.962	1.840	1.692	1.194	0.963	1.229	2.778
**3f**	2.139 (0.2887)	2.105 (0.3086)	2.018 (0.3518)	2.134 (0.3038)	1.890	3.439	1.863	1.650	1.191	0.966	1.224	1.976
**3g**	2.123 (0.3220)	2.088 (0.3381)	1.993 (0.3606)	2.004 (0.3417)	1.896	2.097	1.855	1.698	1.197	0.969	1.222	2.085
**4a**	2.265 (0.2495)	2.235 (0.2698)	2.120 (0.3301)	2.257 (0.2661)	2.060	3.752	2.052	1.790	1.172	0.973	1.234	1.809
**4b**	2.245 (0.2572)	2.168 (0.2957)	2.122 (0.3274)	2.219 (0.2854)	2.031	3.761	1.997	1.778	1.192	0.971	1.225	1.832
**4c**	2.323 (0.2450)	2.280 (0.2693)	2.151 (0.3253)	2.343 (0.2512)	2.048	3.810	2.016	1.786	1.188	0.970	1.225	1.839
**4d**	2.336 (0.2423)	2.290 (0.2663)	2.159 (0.3194)	2.388 (0.2366)	2.056	3.833	2.020	1.788	1.192	0.970	1.220	1.837
**4e**	2.162 (0.3313)	2.098 (0.3528)	2.070 (0.3814)	2.058 (0.3826)	1.984	2.160	1.952	1.821	1.195	0.974	1.232	1.983
**4f**	2.182 (0.2965)	2.168 (0.3082)	2.143 (0.3387)	2.151 (0.3191)	2.013	2.281	1.967	1.814	1.204	0.971	1.211	1.995
**4g**	2.229 (0.3124)	2.195 (0.3307)	2.110 (0.3574)	2.115 (0.3441)	2.044	2.422	1.997	1.837	1.191	0.973	1.226	1.913

Table S3. Selected interatomic distances (Å) in the reagents, prereaction complexes, transition states, and products of the reactions of hypoatranes **5a-g**, **6a-g** (**a** OH, **b** F, **c** Cl, **d** Br, **e** $OClO_3$, **f** ONO_2, **g** SCN) with methanol. For X–N distances the Mayer bond order in the NAO basis are given in parentheses.

	$X–N^{reagent}$	$X–N^{complex}$	$X–N^{TS}$	$X–N^{product}$	$X–O1^{TS}$	$X–O1^{product}$	$X–O2^{TS}$	$X–O2^{product}$	$O1–H3^{TS}$	$O1–H3^{product}$	$O2–H3^{TS}$	$O2–H3^{product}$
**5a**	2.099 (0.3012)	2.114 (0.2951)	2.049 (0.3471)	2.072 (0.3248)	1.929	3.740	1.904	1.690	1.198	0.973	1.218	1.785
**5b**	2.138 (0.2813)	2.046 (0.3416)	2.033 (0.3528)	2.042 (0.3385)	1.891	2.116	1.863	1.724	1.197	0.971	1.228	2.019
**5c**	2.172 (0.2939)	2.041 (0.3579)	2.037 (0.3631)	2.033 (0.3547)	1.887	2.128	1.866	1.718	1.201	0.970	1.221	2.050
**5d**	2.158 (0.2996)	2.081 (0.3425)	2.036 (0.3557)	2.028 (0.3476)	1.886	2.133	1.865	1.712	1.204	0.970	1.218	2.083
**5e**	2.021 (0.3981)	1.967 (0.4346)	1.965 (0.4429)	1.956 (0.4364)	1.838	1.969	1.810	1.684	1.220	0.967	1.220	2.299
**5f**	2.037 (0.3618)	1.969 (0.4119)	1.968 (0.4179)	1.958 (0.4125)	1.863	2.086	1.828	1.684	1.203	0.966	1.226	2.287
**5g**	2.026 (0.3861)	1.988 (0.4034)	1.991 (0.4040)	1.990 (0.3924)	1.873	2.080	1.832	1.700	1.200	0.971	1.227	2.038
**6a**	2.175 (0.3004)	2.162 (0.3213)	2.135 (0.3414)	2.152 (0.3246)	2.049	3.757	2.016	1.808	1.199	0.974	1.220	1.786
**6b**	2.168 (0.3060)	2.121 (0.3388)	2.107 (0.3519)	2.123 (0.3335)	2.005	2.225	1.967	1.826	1.200	0.972	1.229	2.030
**6c**	2.286 (0.2773)	2.159 (0.3419)	2.149 (0.3513)	2.154 (0.34010	2.025	2.307	2.000	1.842	1.200	0.972	1.222	2.014
**6d**	2.307 (0.2693)	2.173 (0.3323)	2.159 (0.3417)	2.161 (0.3317)	2.034	2.348	2.009	1.845	1.201	0.971	1.220	2.021
**6e**	2.089 (0.4084)	2.054 (0.4347)	2.048 (0.4455)	2.042 (0.4381)	1.956	2.105	1.920	1.795	1.217	0.969	1.225	2.226
**6f**	2.119 (0.3613)	2.063 (0.4047)	2.059 (0.4134)	2.050 (0.4087)	1.983	2.209	1.944	1.800	1.202	0.967	1.230	2.256
**6g**	2.168 (0.3689)	2.116 (0.3943)	2.113 (0.3998)	2.119 (0.3845)	2.020	2.270	1.980	1.831	1.200	0.972	1.225	2.008

Table S4. Selected interatomic distances (Å) in the reagents, prereaction complexes, transition states, and products of the reactions of atranes **1a-d**, **2a-d** (**a** OH, **b** F, **c** Cl, **d** Br) with ethanol.

	X–N^{reagent}	X–N^{complex}	X–N^{TS}	X–N^{product}	X–O1^{TS}	X–O1^{product}	X–O4^{TS}	X–O4^{product}	O1–H1^{TS}	O1–H1^{product}	O4–H1^{TS}	O4–H1^{product}
**1a**	2.463	2.446	2.160	2.954	1.953	3.976	1.907	1.639	1.195	0.973	1.208	1.826
**1b**	2.344	2.323	2.121	2.536	1.922	3.686	1.912	1.658	1.185	0.972	1.221	1.816
**1c**	2.382	2.357	2.137	2.691	1.916	3.732	1.912	1.650	1.195	0.970	1.210	1.849
**1d**	2.378	2.351	2.135	2.714	1.914	3.743	1.910	1.649	1.199	0.970	1.206	1.860
**2a**	2.343	2.319	2.253	2.437	2.091	3.712	2.029	1.793	1.197	0.976	1.205	1.762
**2b**	2.280	2.259	2.213	2.328	2.065	3.721	2.030	1.790	1.179	0.976	1.224	1.763
**2c**	2.315	2.289	2.237	2.405	2.071	3.741	2.052	1.793	1.183	0.974	1.218	1.777
**2d**	2.322	2.295	2.242	2.426	2.074	3.747	2.059	1.794	1.185	0.974	1.215	1.782

Table S5. Selected interatomic distances (Å) in the reagents, prereaction complexes, transition states, and products of the reactions of ocanes **3a-d**, **4a-d** (**a** OH, **b** F, **c** Cl, **d** Br) with ethanol.

	X–N^{reagent}	X–N^{complex}	X–N^{TS}	X–N^{product}	X–O1^{TS}	X–O1^{product}	X–O3^{TS}	X–O3^{product}	O1–H2^{TS}	O1–H2^{product}	O3–H2^{TS}	O3–H2^{product}
**3a**	2.271	2.225	2.036	3.088	1.991	4.060	1.924	1.643	1.176	0.969	1.230	2.034
**3b**	2.262	2.205	2.043	2.202	1.921	3.672	1.890	1.660	1.189	0.969	1.224	1.884
**3c**	2.344	2.253	2.050	2.043	1.911	2.204	1.883	1.733	1.195	0.973	1.220	1.916
**3d**	2.318	2.233	2.044	2.033	1.909	2.172	1.877	1.729	1.202	0.973	1.213	1.935
**4a**	2.265	2.213	2.158	2.226	2.081	3.753	2.012	1.796	1.215	0.976	1.198	1.760
**4b**	2.245	2.163	2.163	2.187	2.029	2.295	1.976	1.833	1.206	0.974	1.216	1.943
**4c**	2.323	2.259	2.198	2.213	2.040	2.453	2.005	1.838	1.215	0.971	1.201	1.958
**4d**	2.336	2.270	2.205	2.303	2.044	3.763	2.017	1.794	1.214	0.973	1.200	1.800

Table S6. Selected interatomic distances (Å) in the reagents, prereaction complexes, transition states, and products of the reactions of hypoatranes **5a-d**, **6a-d** (**a** OH, **b** F, **c** Cl, **d** Br) with ethanol.

	X–N^{reagent}	X–N^{complex}	X–N^{TS}	X–N^{product}	X–O1^{TS}	X–O1^{product}	X–O2^{TS}	X–O2^{product}	O1–H3^{TS}	O1–H3^{product}	O2–H3^{TS}	O2–H3^{product}
**5a**	2.099	2.092	2.008	2.098	1.991	3.689	1.910	1.681	1.185	0.971	1.222	1.843
**5b**	2.138	2.047	2.034	2.042	1.892	2.119	1.860	1.724	1.196	0.971	1.230	2.018
**5c**	2.171	2.039	2.038	2.032	1.889	2.138	1.862	1.717	1.199	0.970	1.224	2.057
**5d**	2.158	2.080	2.036	2.027	1.888	2.144	1.860	1.717	1.203	0.969	1.221	2.094
**6a**	2.175	2.161	2.094	2.127	2.104	3.839	2.029	1.810	1.191	0.974	1.221	1.857
**6b**	2.168	2.121	2.108	2.124	2.006	2.228	1.964	1.826	1.200	0.972	1.231	2.027
**6c**	2.286	2.159	2.150	2.155	2.026	2.313	1.996	1.842	1.199	0.972	1.224	2.012
**6d**	2.308	2.172	2.159	2.161	2.035	2.357	2.005	1.843	1.199	0.971	1.222	2.024
